# A Gene-Environment Study of Cytoglobin in the Human and Rat Hippocampus

**DOI:** 10.1371/journal.pone.0063288

**Published:** 2013-05-16

**Authors:** Christian Ansgar Hundahl, Betina Elfving, Heidi Kaastrup Müller, Anders Hay-Schmidt, Gregers Wegener

**Affiliations:** 1 Centre of Excellence for Translational Medicine, University of Tartu, Tartu, Estonia; 2 Translational Neuropsychiatry Unit, Department of Clinical Medicine, Aarhus University, Aarhus, Denmark; 3 Department of Neuroscience and Pharmacology, The Panum Institute, University of Copenhagen, Copenhagen, Denmark; 4 Unit for Drug Research and Development, School of Pharmacy (Pharmacology), North-West University, Potchefstroom, South Africa; University of Regensburg, Germany

## Abstract

**Background:**

Cytoglobin (Cygb) was discovered a decade ago as the fourth vertebrate heme-globin. The function of Cygb is still unknown, but accumulating evidence from *in vitro* studies point to a putative role in scavenging of reactive oxygen species and nitric oxide metabolism and *in vivo* studies have shown Cygb to be up regulated by hypoxic stress. This study addresses three main questions related to Cygb expression in the hippocampus: 1) Is the rat hippocampus a valid neuroanatomical model for the human hippocampus; 2) What is the degree of co-expression of Cygb and neuronal nitric oxide synthase (nNOS) in the rat hippocampus; 3) The effect of chronic restraint stress (CRS) on Cygb and nNOS expression.

**Methods:**

Immunohistochemistry was used to compare Cygb expression in the human and rat hippocampi as well as Cygb and nNOS co-expression in the rat hippocampus. Transcription and translation of Cygb and nNOS were investigated using quantitative real-time polymerase chain reaction (real-time qPCR) and Western blotting on hippocampi from Flinders (FSL/FRL) rats exposed to CRS.

**Principal Findings:**

Cygb expression pattern in the human and rat hippocampus was found to be similar. A high degree of Cygb and nNOS co-expression was observed in the rat hippocampus. The protein levels of nNOS and Cygb were significantly up-regulated in FSL animals in the dorsal hippocampus. In the ventral hippocampus Cygb protein levels were significantly up-regulated in the FSL compared to the FRL, following CRS.

**Significance:**

The rodent hippocampus can be used to probe questions related to Cygb protein localization in human hippocampus. The high degree of Cygb and nNOS co-expression gives support for Cygb involvement in nitric oxide metabolism. CRS induced Cygb and nNOS expression indicating that Cygb expression is stress responsive. Cygb and nNOS may be important in physiological response to stress.

## Introduction

Cytoglobin (Cygb) was discovered as the forth vertebrate heme-globin baring structural resemblance to the well known hemoglobin and myoglobin in spite of low sequence homology [1]–[3]. Cygb can reversibly bind oxygen and other diatomic gases with an affinity similar to that of myoglobin [4], [5] and is expressed in most tissues investigated [6]–[8]. Of particular interest, Cygb was also found in neurons of the brain in both the cell soma and nucleus [6]–[8]. In the rodent brain Cygb mRNA and protein is expressed in well-defined brain areas, but with profound differences in expression levels [6], [7], [9], with the highest Cygb expression in the reticular thalamic nucleus, habenula nucleus, hippocampus, laterodorsal- and pedunculopontine tegmentalnucleus, and several hypothalamic nuclei [7], [9]. Despite intense research, the function of Cygb remains to be established. However, some studies have shown that Cygb may be involved in neuronal protection against reactive oxygen species (ROS) mediated damage and Cygb expression can be up-regulated by hypoxic stress (for review see Burmester et al. 2007 [10]). Therefore, the high expression of Cygb in the hippocampus may be of significant relevance, since the hippocampus is one of the areas of the brain prone to the adverse effects of stress. Importantly, changes in this brain structure are seen in depression, schizophrenia, and neurological diseases such as Alzheimer’s disease, disorders where oxidative stress has been suggested to be a major pathological factor (for review see references [11]–[13]). Among the stress factors other than ROS, are nitric oxide (NO) and its metabolites, which have been shown to be up-regulated in the hippocampi in animal models of depression and stress [14]–[19] , and in postmortem human studies [20], [21].

Interestingly, we have recently shown most neuronal nitric oxide synthase (nNOS) positive neurons in the mouse brain to co-express Cygb [7], and there is evidence that Cygb may regulate the levels of free NO *in vitro* by functioning as a dioxygenase [22]–[24] and recently reviewed by Gardner [25]. This combined information can be hypothesized to reflect a physiological role of Cygb in regulating nNOS/NO release or production and therefore also the physiological effects mediated by NO. Given the possible impact of NO and ROS in disorders with disturbed hippocampal function, and the ROS and NO scavenging properties of Cygb, the aim of the present study was to describe the anatomical distribution of Cygb protein in the human and rat hippocampus and in a relevant gene-environmental animal model of disease, the Flinders Sensitive Rat combined with environmental stress.

## Results

### Cygb-immuno Reactivity (IR) in the Human and Rat Hippocampus (Figure 1 and 2)

**Figure 1:**
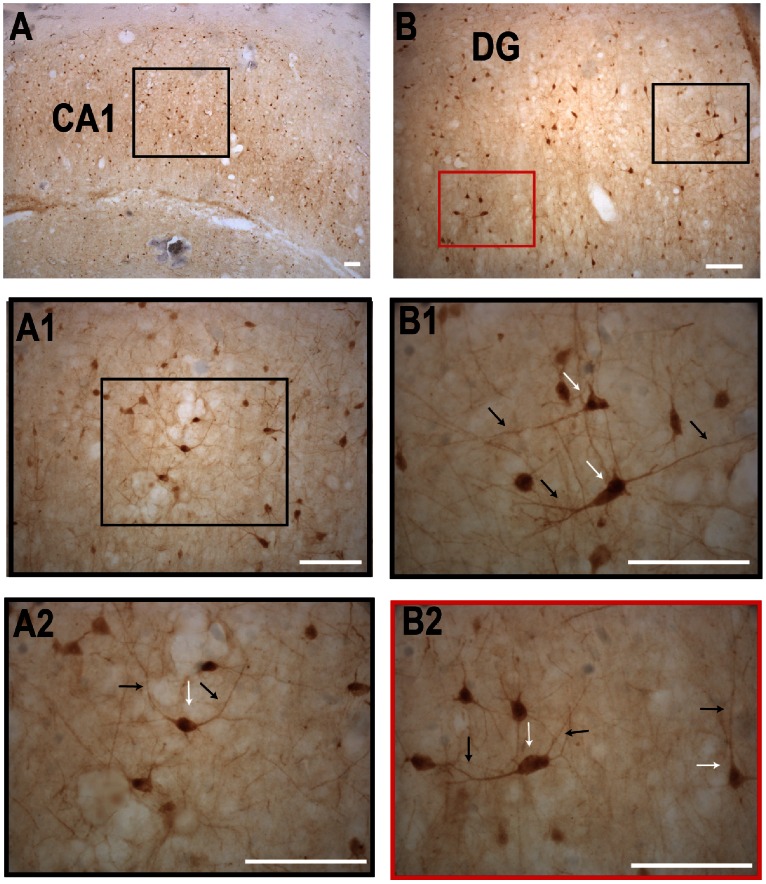
Cygb immunoreactivity in the human hippocampus. **A** Overview of the human hippocampus. **A1–A2** shown higher magnifications of the area in the black square. Note intense expression of Cygb-IR in both the cell soma (white arrow) and processes (black arrow). **B** and **B1–2** shows Cygb-IR in the dente gyrus (DG). Scale bar 100 µm.

**Figure 2:**
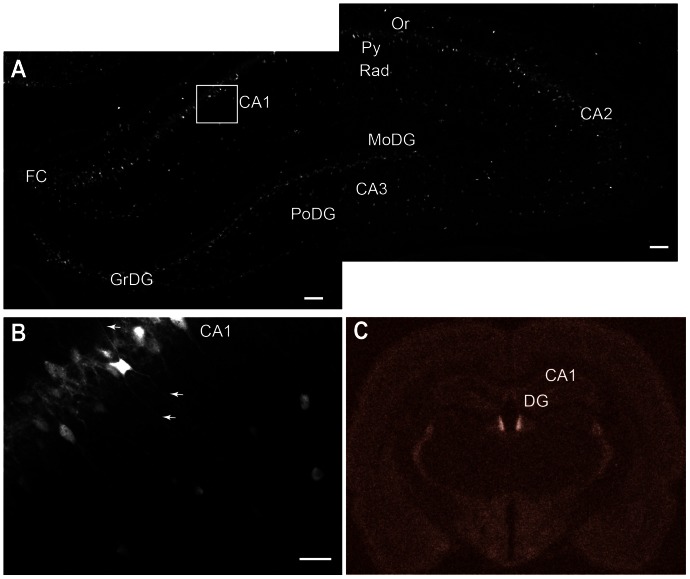
Cygb immunoreactivity and mRNA expression in the rat hippocampus. **A** Overview of Cygb-IR in the rat hippocampus. High levels of Cygb-IR can be seen in most layers of the hippocampus. The areas within the white square is magnified in **B** showing Cygb-IR in cell bodies and there processes, marked by white arrows, of CA1 neurons. In **C** an *in situ hybridization* of Cygb mRNA expression is shown in the rat hippocampus. Abbreviations: Cornu ammonis1–3 (CA1–3), dentate gyrus (DG), fascio larumcinereum (FC), striatum granulosum of DG (GrDG), molecular layer of DG (MoDG), oriens layer of the hippocampus (Or), polymorph layer of DG (PoDG), pyramidal cell layer (Py), stratum radiatum (Rad). Scale bar 100 µm.

In the human hippocampus Cygb-IR was seen dispersed throughout the CA1–CA3 and dentate gyrus (DG) in medium to large sized neurons in the cytoplasm and processes, forming a dense network of Cygb-IR fibers (Fig. 1A–B). Similarly, high levels of Cygb-IR could be seen in the rat hippocampus from Bregma level −1.72 to −6.84 [26] (Fig. 2A). Cygb-IR was seen in field CA1–CA3 of hippocampus (Fig. 2A–B). Cygb-IR cell bodies were in both the pyramidal cell layer (Py) and stratum radiatum (Rad) of the hippocampus (Fig. 2A). Processes from all layers were both descending and ascending. High expressing cell bodies and their processes were also seen in DG in both the molecular layer of DG (MoDG) and polymorph layer of DG (PoDG) (Fig. 2A). The Cygb-IR expression pattern was confirmed by *in situ* hybridization (ISH) showing a strong signal in the DG and moderate signal in CA1–CA3 (Fig. 2C).

### Cygb-IR and nNOS-IR Co-expression in Rat Hippocampus (Figure 3, 4, 5)

**Figure 3:**
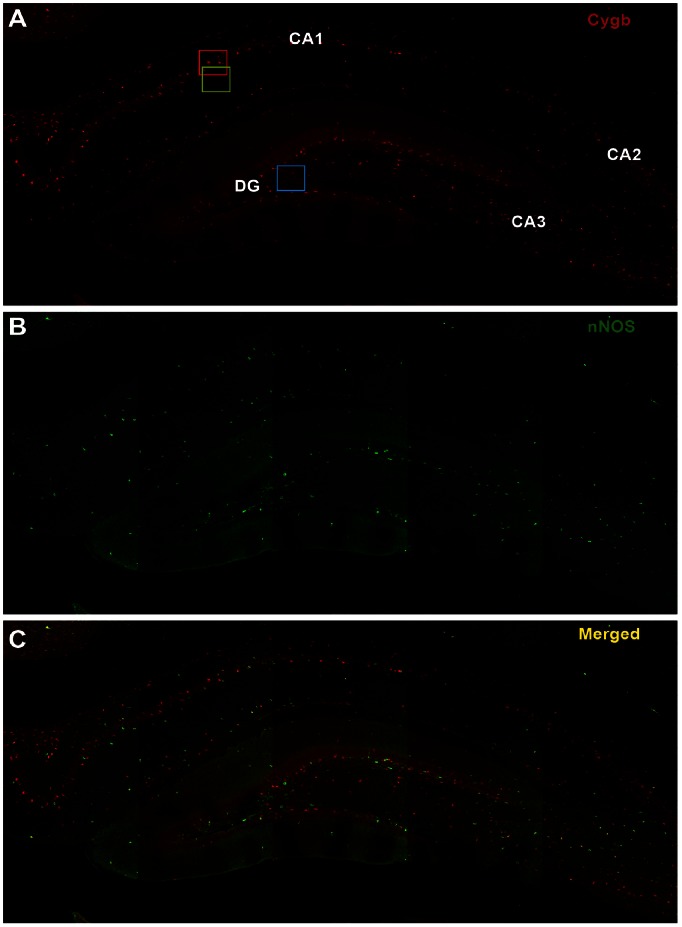
Overview of Cygb and nNOS expression and co-expression in the hippocampus. **A** shows an overview of Cygb-IR (red) in the rat hippocampus. Red, green and blue squares are shown in higher magnification in figure 4. **B** shows an overview of nNOS-IR (green) expression and **C** is the merged image of **A** and **B.** Abbreviations: Cornu ammonis1–3 (CA1–3), dentate gyrus (DG).

**Figure 4:**
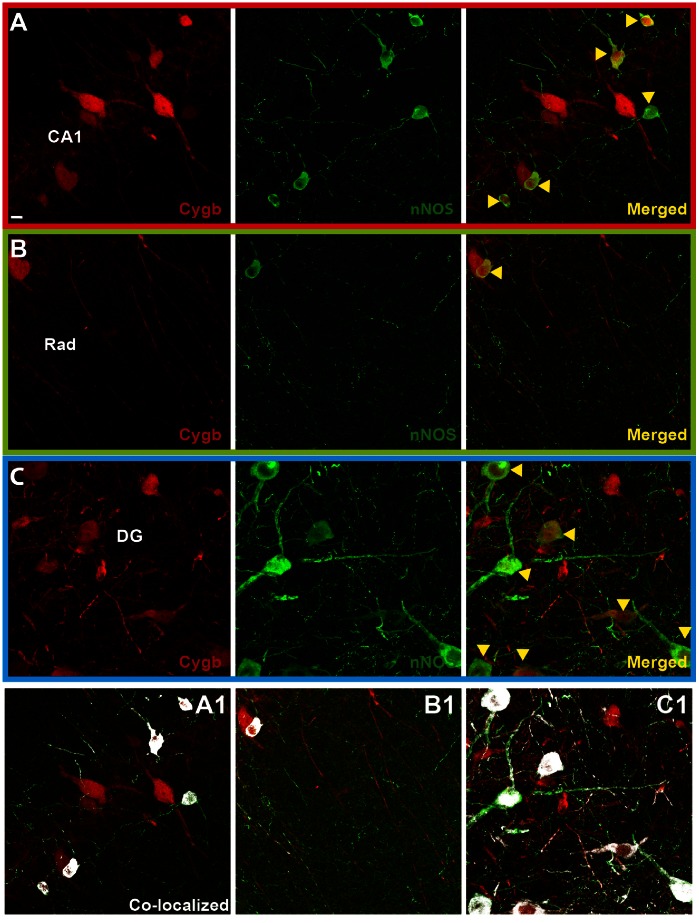
Analysis of Cygb and nNOS co-localization. **A–C** shows high-resolution stacks of the areas from figure 3. **A** Cygb-IR (red) and nNOS-IR (green) in the CA1. Yellow arrowheads denote neurons with co-localized Cygb-IR and nNOS-IR. **B** shows the stratum radiatum (Rad). Note most nNOS-IR fibers do not co-express Cygb-IR. **C** shows the dente gyrus (DG). A1–C1 shows calculated co-localization (white). Scale bar 50 µm.

**Figure 5:**
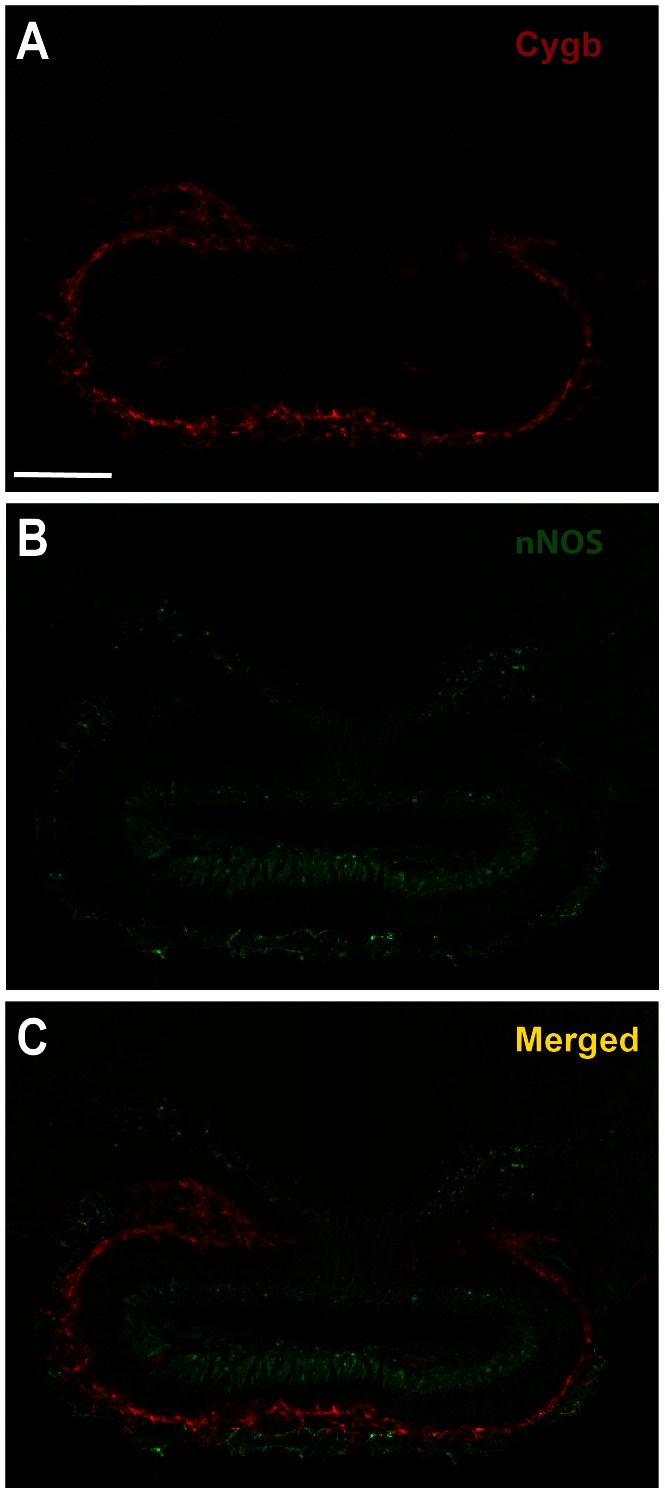
Validation of the immunohistochemistry method. **A** and **B** shows Cygb-IR (red) and nNOS-IR (green), respectively, in a coronal section of the rat pituitary gland. **C** is the merged image of **A** and **B**. Note complete separation of the red and green signal showing no false cross reactivity from the secondary antibodies or artifact due to problematic filter settings of the microscope. Scale bar 50 µm.

In comparison to nNOS-IR, Cygb-IR was more numerous in all areas of the hippocampus (Fig. 3A–C). High magnification images of the areas marked by boxes in Fig. 3 shows that most of the nNOS-IR neuronal somas were found to co-store Cygb-IR whereas parts of the dense nNOS-IR fiber network seen throughout the hippocampus did not (Fig. 4A–C). This observation was supported by image analysis of the degree of co-localization (Fig. 4A1–C1). The observed high degree of co-localization could be an artifact of the staining method. However, double staining of Cygb-IR and nNOS-IR in the pituitary gland show clear separation of Cygb-IR and nNOS-IR verifying that the observed co-expression was not due to cross reaction of the secondary antibodies or problematic microscope filter settings (Fig. 5).

### Cygb and nNOS Gene Expression in the Hippocampus Following CRS (Figure 6)

Having established the rat hippocampus to have high expression levels of Cygb, and the co-storage of Cygb and nNOS in most nNOS neurons, it was investigated whether Cygb gene expression may be regulated by stress, and if so whether changes may happen concomitantly with a regulation of the nNOS gene expression.

**Figure 6:**
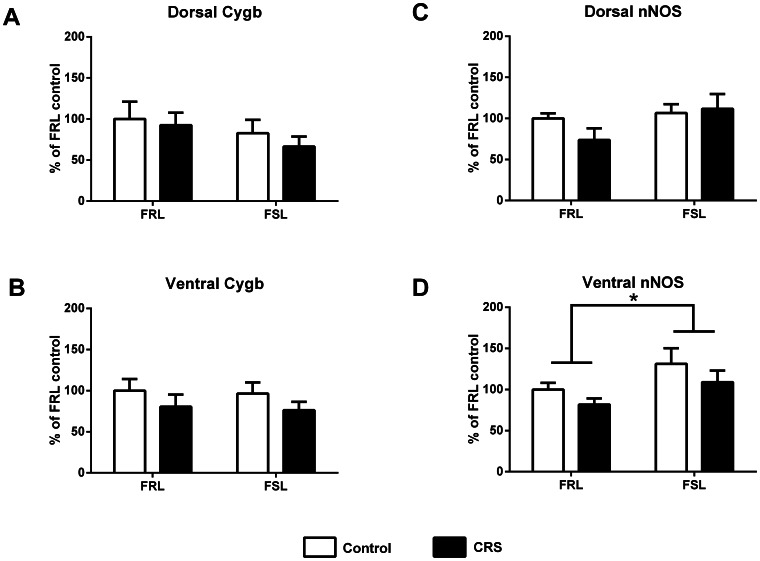
mRNA expression of Cygb and nNOS. mRNA expression of Cygb and nNOS in the dorsal and ventral hippocampus after chronic restraint stress (CRS) using real-time qPCR. Values for each individual were normalized with the geometric mean of the reference genes CycA/Actb and CycA/Hprt1 for the dorsal and ventral hippocampus, respectively. Plotted data show mean group values+SEM of mRNA expression as % of FRL control. Differences between groups were analyzed using two-way ANOVA (*p<0.05, between FSL and FRL strains). n = 6 in each group.

In the dorsal/ventral hippocampus no strain and stress effects were found on the Cygb expression (Fig. 6A–B). However, the overall Cygb levels had a tendency of being lower in the FSL rats, especially in the dorsal hippocampus (Fig. 6A). In contrast, the nNOS expression was significantly up-regulated in the ventral hippocampus in the FSL rats compared to the FRL rats (F(1,20) = 5.02, p = 0.0365), but no interaction with CRS was observed on nNOS expression (Fig. 6C–D).

### Cygb and nNOS Protein Expression in the Hippocampus Following CRS (Figure 7 and 8)

**Figure 7:**
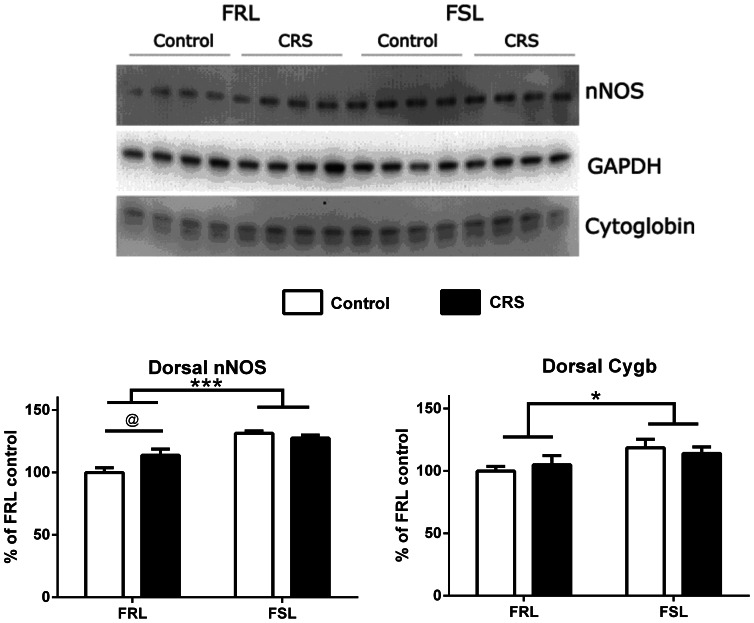
Western blot analysis of Cygb and nNOS in dorsal hippocampus after CRS. Representative blots are shown. Graphs represent the statistical densitometric analyses of data from three separate experiments. Data are expressed as mean group values+SEM of FRL control. Two-way ANOVA analysis revealed differences between FRL versus FSL (*p<0.05; ***p<0.001, both between FSL and FRL strains) and a significant effect of CRS in the FRL rats (*p<0.05; two-way ANOVA followed by Bonferroni's multiple comparison test).

**Figure 8:**
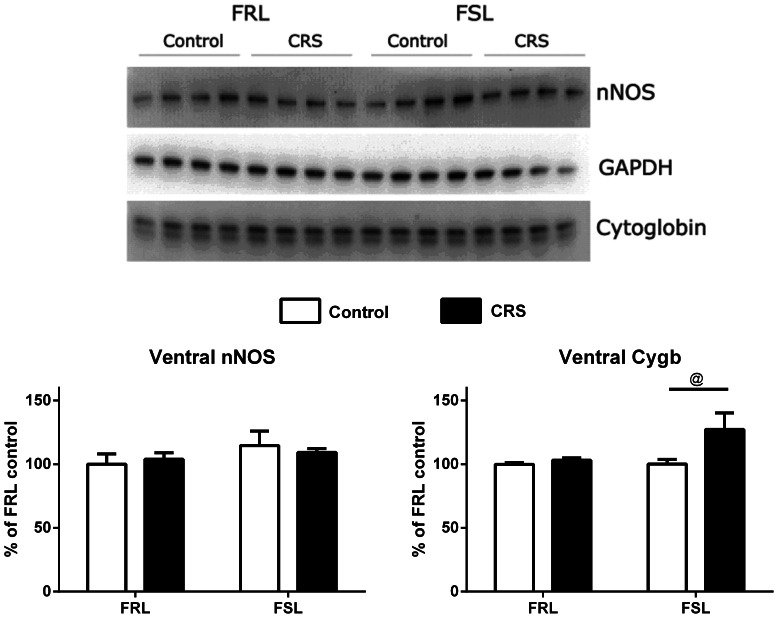
Western blot analysis of Cygb and nNOS in ventral hippocampus after CRS. Western blot analysis of Cygb and nNOS in ventral hippocampus after CRS. Representative blots are shown. Graphs represent the statistical densitometric analyses of data from three separate experiments. Data are expressed as mean group values+SEM of FRL control. Two-way ANOVA analysis revealed differences a significant effect of CRS in the FSL (*p<0.05; two-way ANOVA followed by Bonferroni's multiple comparison test).

In the dorsal hippocampus (Fig. 7), higher levels of Cygb and nNOS were observed in the FSL rats compared to the FRL rats (F(1,12) = 5.40, p = 0.0386; F(1,12) = 41.87, p<0.0001, respectively). For nNOS, a significant interaction was present, and bonferronís post-hoc test revealed a significant effect of CRS, in that nNOS was up-regulated in the FRL rats (p = 0.0273). In the ventral hippocampus (Fig. 8), only minor changes were observed. Using two-way ANOVA, we found that Cygb was significantly up-regulated after CRS in the ventral hippocampi of the FSL rats (F(1,12) = 4.95, p = 0.0461, post-hoc bonferroni p<0.05) even though the variation was due to stress only, and no interaction between strain and stress was found.

## Discussion

A considerable number of studies have evaluated Cygb expression and possible neuroprotective effects during hypoxia, ischemia and ROS mediated stress [10], [27], [28], and a possible Cygb functional link to the nNOS/NO system [7], [22], [23]. However, no studies have to our knowledge investigated the Cygb expression in the human hippocampus or in an animal stress model known to produce increased levels of NO and ROS in order to resolve if Cygb gene/protein expression is affected by stress and whether nNOS/NO expression changes co-occur with a change in Cygb expression *in vivo.*

The main finding in this paper is the demonstration that the human Cygb protein expression pattern and the morphology of the Cygb-IR neurons are well in correspondence with what is seen in the rat hippocampus, suggesting that the rodent model at the anatomical level is representative of the human brain. Importantly, the overall expression pattern of Cygb-IR reported in this study for human and rat is also in line with previous work done in mice [7], [9] and is confirmed by ISH in this study. An interesting observation is the very dense network of neuronal processes seen both in the human and rat hippocampus, indicating that Cygb function is not only restricted to the cell soma. We report here an extensive co-expression between Cygb and nNOS in the rat hippocampus, both in the cell soma and processes. These findings extend earlier co-localization studies in the mouse brain [7] thus providing new *in vivo* anatomical basis for the Cygb/NO interactions observed in *in vitro* models [22]–[24]. It should be noted that sparse co-localization of Cygb was seen in the dense nNOS fiber network observed throughout the hippocampus. A likely explanation may be that these fibers are afferent projections to the hippocampus from brain structures where the degree of co-localization between Cygb and nNOS is lower such as the entorhinal cortex, the medial septum/diagonal band, lateral preoptic area and supramammillary nucleus [7], [29].

By the use of a genetic animal model of depression with addition of stress, we aimed at clarifying Cygb related changes following genetic and environmental factors. The co-localization of Cygb and nNOS is of significant interest, as substantial amount of studies indicate involvement of the NO system in stress-related disorders, such as affective disorders. In postmortem material from patients with depression, reduced nNOS containing neurons in the paraventricular hypothalamic nucleus, prefrontal cortex and locus coeruleus [30]–[33], and an increase in CA1 hippocampal area has been reported [20]. Similarly, studies of peripheral markers have reported increased NO metabolites (NO_2_^−^ and NO_3_^−^) [34]–[36], indicating a hyperfunction of the nitrergic system. In the last study, treatment with antidepressants normalized the NO_2_^−^ levels, correlating with clinical response [36]. Furthermore, a recent study showed that Cygb has a potential protective function by reducing NO_2_^−^ to NO and downstream activation of soluble guanylylcyclase in smooth muscle cells, being especially pronounced under low oxygen conditions as would occur following ischemic and/or hypoxic stress [24]. In addition, Cygb oxygen dependent binding of NO was proposed to act as a sensitive transducer of changes in oxygen concentration to a change in NO consumption, thereby allowing Cygb to sense and regulate NO in response to changes in tissue oxygenation [22], [23], [37]. These studies indicate two different functions of Cygb in either NO production from the reduction of NO_2_^−^ to NO or as an NO sink via dioxygenation to NO_3_^−^ governed by the oxygen concentration of the tissue. Together, these studies show that Cygb potentially can have a key role in regulating the level of free NO and thereby NO downstream targets. It is therefore of significance to elaborate upon the potential NO scavenging mechanisms functioning *in vivo,* and the high degree of co-expression between Cygb and nNOS in the rat hippocampus, suggesting a physiological relevance *in vivo*.

We used the well-established FSL and FRL stress susceptible animal model of depression to probe the effect of gene environment stress on Cygb and nNOS expression. On gene level we observed a strain difference only in nNOS expression, which was up-regulated in the ventral hippocampi of FSL, which is in contrast to previous studies where no difference was observed between the two lines [17]. An up-regulation of NO signaling in the FSL rats, not only under stressful conditions, but also in the basal state, may indeed contribute to the psychopathological traits in the FSL model, which can be observed during basal conditions [38]. Contrasting our earlier studies, the present study employed a higher spatial resolution which was obtained by dissecting the hippocampus in a dorsal and ventral part whereas in the former study the whole hippocampus was used, which could have masked changes in regional expression. At the protein level both Cygb and nNOS were higher in the dorsal hippocampus of the FSL rats. Lesion studies have revealed that the dorsal hippocampus is involved in learning and spatial memory, whereas the ventral hippocampus regulates emotional and motivated behaviors [39]. Although the present study shows a discrepancy between gene-expression and protein data, the data presented here support an important role for nNOS in both the ventral and dorsal hippocampus.

We have no clear explanation of the discrepancy between the mRNA expression and the protein levels. As the present work represent a cross-sectional study, a possible explanation may be due to differences in expression in the different regions at different times, and we can here not account for the detailed turnover of each protein/mRNA. Moreover, from earlier studies, it is known that extrapolating the mRNA results to protein results is not possible as correlation analysis in large-scale data sets only reports 50% correspondence between mRNA and protein levels [40]–[42].

CRS is known to induce dendritic atrophy [43], reduce neurogenesis [44], and oxidative damage [45]. Several studies have shown antioxidant properties of Cygb and interestingly Cygb has reactive cystine residues [4] like many of the *bona fide* oxidative stress regulatory proteins. Cygb gene expression was unaffected by CRS, but Cygb protein was up-regulated in the FSL animals in agreement with the higher expression in the naïve animals and in line with studies showing Cygb to be up-regulated by oxidative stress [6], [9], [46]. However, the stress effect was only evident in the genetically sensitive animals. The exact explanation and consequences of this finding remains obscure, and further studies are warranted.

Previously, we have investigated nitrergic signaling in the FSL/FRL animals, showing that no major abnormalities in the nitrergic signaling exist between the sensitive and the vulnerable line in basal state, but the two lines respond differently to an escapable-inescapable stress paradigm – suggesting a dysfunctional nitrergic system following a stressful life-event [17]. We were not able to reproduce this finding in the present work following another stress-paradigm, where the mRNA expression of nNOS in both dorsal and ventral hippocampus was unaffected following CRS and similarly for Cygb. CRS did induce an increase in the protein expression of nNOS in the dorsal hippocampi of the FRL rats. However, as the most robust increase in nNOS protein expression, the dorsal hippocampus, it is not reflected in Cygb indicating that there is no direct correlation between the protein levels of nNOS and Cygb following CRS. The up-regulation of nNOS corroborates previous studies showing an increased nNOS protein expression after CRS [15].

A limitation of the present study is the cross-sectional design, which makes the conclusions based on the expression data more speculative. Future studies should add further time-points in order to assess the longitudinal expression of nNOS and Cygb. Also, we did not take into account the NO contribution arising from inducible NOS (iNOS). It is well known that iNOS may be induced in the brain following chronic stress [47]. Indeed, it was previously shown that the effects following a PTSD stress paradigm could be abolished by an iNOS inhibitor, but not by an inhibitor of nNOS [16], [48].

In conclusion, the rodent hippocampus can be used to probe questions related to Cygb protein localization in human hippocampus. The high degree of Cygb and nNOS co-expression gives neuroanatomical support for Cygb involvement in nitric oxide metabolism.

## Materials and Methods

### Ethics Statement

The use of the human brain material was done in accordance with Danish Ministry of Justice, The Danish National Committee for Ethics (Health Law no. 546, §188). The body was bequeathed to science and education at the Department of Cellular and Molecular Medicine at The University of Copenhagen, and use hereof was approved by the head of the Body Donation Program at the Department of Cellular and Molecular Medicine, University of Copenhagen.

All animal procedures were approved by the Danish National Committee for Ethics in Animal Experimentation (2007/561-1378).

### Human Brain Material

Human brain was donated by “The body donation program”, Panum Institute, University of Copenhagen, Denmark. Perfusion fixation in 10% formalin occurred between 6 to 24 hours after time of death. The specimen used for the localization studies of Cygb in the hippocampus was donated by a 74-year-old woman with an unknown course of death. At autopsy there were no signs of pathological alterations in the brains.

After fixation the brain was sectioned in 1 cm sagittal blocks and cryoprotected in 30% sucrose-PBS for five days, frozen and subsequently sectioned in 40 µm thick coronal slices.

### Animals

Male FSL and FRL rats (age 10–12 weeks), from the colony maintained at the Aarhus University were used. All rats weighed 280–350 g prior to experiments, and were cage-housed individually (Cage 1291H Eurostandard Type III H, 425×266×185 mm, Techoplast, Italy) at 20±2°C in a 12 h light/dark cycle (lights on at 7.00 a.m.). Tap water and chow pellets were available *ad libitum*. The animal colony was protected from outside noise, and all experimental procedures were performed in specially-equipped rooms within the animal house.

The stress protocol was carried out as previously described [49],[50], with minor modifications. Briefly, the rats were randomly and evenly divided into a group receiving exposure to stress and a control group. During the two week stress period, the control rats remained in their home cages with daily handling. In a separate room, the stress group was subjected to a 2 hour daily restraint stress schedule (09.00 a.m. to 11.00 a.m.) for 14 days in transparent acrylic restrainers secured at the head and tail, with an intensive light source above (1000 Lux).

For the detailed histological characterization of Cygb-IR, four 250 g Wistar male rats (Taconic, Denmark) were used. The rats were housed under standard 12∶12 light:dark conditions (lights on at 06∶00) with food/water *ad libitum*.

### Tissue Preparation

All animals were euthanized within the same time window each day (11–14∶00 hours). Stressed animals were euthanized exactly 2 hours following completion of the stress paradigm. Following decapitation the brains were removed and divided into two hemispheres. One hemisphere was put in phosphate buffered 4% paraformaldehyde (PFA) pH 7.2. Hippocampus from the other hemisphere was rapidly dissected, and immediately frozen in dry-ice powder and stored at −80 degrees until further analysis. After immersion fixation of the rat brains in 4% PFA for several days, the hemispheres were cryoprotected in 30% sucrose-PBS for 5 days, frozen and sectioned in 40 µm thick coronal slices in replicas of five.

For the histological study, Wistar rats were anaesthetized using a solution of pentobarbital/lidocain, followed by perfusion fixation with phosphate buffered 4% PFA fixative pH 7.2. Thereafter, the brains were removed and post-fixed in the same fixative overnight, cryoprotected in 30% sucrose-PBS for 5 days, frozen and sectioned in 40 µm thick coronal slices in replicas of five.

### Immunohistochemistry

For detection of Cygb-IR two in house made antibodies raised in rabbits and guinea pigs were used, which were characterized previously [7]. For detection of nNOS a polyclonal rabbit antibody (Enzo Life Sciences, code# ALX-210-501, AH Diagnostics AS, Denmark) was used. The immunostaining was performed as described in [51], [52]. A high degree of autofluorescence was observed in the human brain material making fluorescence detection of Cygb impossible. To overcome this problem diaminobenzidine (DAB) was used. For single free floating immunostaining sections were incubated over night with rabbit anti-Cygb (code no: 5092/6, diluted 1∶10.000) and detected with a biotinylated donkey anti-rabbit (Fab)_2_ (code no: 711-066-152 Jackson Immunoresearch Laboratories, Baltimore, PA, USA, diluted 1∶2000) in combination with Avidin-Biotin-peroxidase Complex (ABC) (VWR international, Roedovre Denmark), followed by 0.05% DAB. For double immunostaining in the rat brain, sections were incubated over night with a guinea pig anti-Cygb (code no: 12168/7, diluted 1∶3000) in combination rabbit anti-nNOS diluted 1∶2000. The primary antibodies were detected with a donkey anti-guinea pig (Fab)_2_ conjugated with Dylight-594 (code#: 706-516-148 Jackson Immunoresearch Laboratories, Baltimore, PA, USA, diluted 1∶500) and donkey anti-rabbit (Fab)_2_ conjugated with Dylight-488 (code no: 706-486-152 Jackson Immunoresearch Laboratories, Baltimore, PA, USA, diluted 1∶500).

### *In situ* Hybridization

A 39′mer DNA oligonucleotide probe complementary to mouse Cygb mRNA (gi:146149214) bases 599-561 5′-gatcctccatgtgtctaaactggctgaagtactgcttgg-3′ was labeled with ^33^P-dATP (PerkinElmer, Europe) using terminal transferase (code no: 3333566, Roche, Denmark) and used for free floating ISH detection of Cygb as described in [7]. In brief, PFA fixed sections of rat brain were acetylated and incubated in hybridization buffer for 1 hour at 38°C. 500.000 cpm/ml were added to the hybridization buffer and the sections were incubated over night on a shaking table at 38°C. Sections were washed 4×15 min at 55°C in 1X saline sodium citrate (SSC) followed by 2×15 min in 0.1X SSC at room temperature. The sections were rinsed in MilliQ water, mounted on glass slides and exposed on a Kodak MR x-ray film (Kodak, Denmark) for two weeks at 4°C.

### Microscopy

Photomicrographs were obtained using a Zeiss LSM780 confocal microscopy equipped with appropriate filter settings for detecting 488 and 568 flourophores. Images were processed in Adobe Photoshop CS5.

### nNOS Innervations of Cygb Expressing Neurons

Analysis of contacts between nNOS-containing fibers and Cygb expressing fibers and neurons was performed using high-resolution stacks of images obtained from 40-µm sections of the hippocampus. Stacks consisted of approximately 25 images with a thickness of approximately 0.4-µm equal to a scan depth of approximately 11-µm. Laser intensity was adjusted within the borders of the gray tone scale. Image analysis was performed using the open-source image program Fiji [53] as described in Hundahl et al 2012 [54].

### Real-time qPCR and Western Blotting

The ParisTM RNA and protein isolation kit (Ambion, TX, USA) was used to isolate RNA and protein from dorsal/ventral hippocampus. The isolation was processed according to the manufacturer’s specifications and as described previously by Müller et al, [55].

#### Real-time qPCR

After RNA extraction the integrity of RNA and the RNA concentration was determined with RNA StdSens microfluidic chips using the Experion Automated Electrophoresis System (BIORAD, CA, USA). The RNA purity and the RNA concentration were determined by spectrophotometer (Nanodrop 1000, Thermo Scientific, MA, USA). Data on quality and purity of the extracted RNA was evaluated by one-way ANOVA.

Before cDNA synthesis the RNA concentration of the samples (n = 48) was adjusted to match the sample with the lowest concentration. RNA was reversely transcribed using random primers and Superscript III Reverse Transcriptase (Invitrogen, CA, USA) following manufactureŕs instructions and with a RNA concentration of 35 ng/µl. The cDNA samples were diluted 1∶30 with DEPC water before being used as a qPCR template.

Real-time qPCR was carried out in 96-well PCR-plates using the Mx3000P (Stratagene, USA) and SYBR Green as previously described [56], [57]. The gene expression of eight different reference genes, nNOS and Cygb were investigated (see Table 1). The reference genes were selected as previously described [58]. The primers were designed and tested prior to use as described previously [56]. Each SYBR Green reaction (10 µl total volume) contained 1× SYBR Green master mix (BIORAD, CA, USA), 0.5 µM primer pairs, and 3 µl of diluted cDNA. All samples were run in duplicate. A standard curve, performed in duplicate, was generated on each plate. Essential gene specific data on primer sequence and amplicon sizes is given in Table 1. The primers were obtained from DNA Technology A/S, Denmark.

**Table 1:** Characteristics of gene-specific real-time qPCR primers.

*Gene Symbol*	*Gene Name*	*Accession No.* 1	*Primer Sequence*	*Amplicon size* 2
**Reference genes**				
18 s rRNA	18 s subunit ribosomal RNA	M11188	(+) acggaccagagcgaaagcat(−) tgtcaatcctgtccgtgtcc	310
ActB	Beta-actin	NM_031144	(+) tgtcaccaactgggacgata(−) ggggtgttgaaggtctcaaa	165
CycA	Cyclophilin A	XM_345810	(+) agcactggggagaaaggatt(−) agccactcagtcttggcagt	248
Gapd	Glyceraldehyde-3-phosphate dehydrogenase	NM_017008	(+) tcaccaccatggagaaggc(−) gctaagcagttggtggtgca	168
Hmbs	Hydroxy-methylbilane synthase	NM_013168	(+) tcctggctttaccattggag(−) tgaattccaggtgagggaac	176
Hprt1	Hypoxanthine guanine phosphoribosyl- transferase	1NM_012583	(+) gcagactttgctttccttgg(−) cgagaggtccttttcaccag	81
Rpl13A	Ribosomal protein L13A	NM_173340	(+) acaagaaaaagcggatggtg(−) ttccggtaatggatctttgc	167
Ywhaz	Tyrosine 3-monooxygenase/tryptophan 5-monooxygenase activation protein, zeta	BC094305	(+) ttgagcagaagacggaaggt(−) gaagcattggggatcaagaa	136
**Target genes**				
Cygb	Cytoglobin	NM_130744	(+) tctctctggggtcattctgg(−) gggcagtgtggctggtag	181
nNOS	Neuronal Nitric Oxide Synthase	NM_052799	(+) accccgtcctttgaatacca(−) acgctgttgaatcggacctt	455

1 Genbank accession number of cDNA and corresponding gene, available at http://www.ncbi.nlm.nih.gov/.

2 Amplicon length in base pairs.

#### Western blotting

Aliquots of total homogenate from individual animals were diluted in lysis buffer (50 mM Tris-HCl, pH 7.4, 150 mM NaCl, 1% Triton X-100, 0.1% SDS, 2 mM EDTA, 0.1 mM EGTA, 5 mM NaF, 1 mM Na_3_VO_4_, 5 mM Na_2_PO_4_ and 1×proteinase inhibitor cocktail (Roche, Mannheim, Germany)) to a final protein concentration of 2 µg/µl. The Western blotting was conducted and quantified as described in [42]. The following antibodies were used: rabbit anti-Cygb 1∶1000 (5092/6, in house), rabbit anti-nNOS 1∶500 (Santa Cruz, sc-546) and mouse anti-GAPDH 1∶2000 (Covance) followed by the appropriate HPP-conjugated secondary antibodies: anti-rabbit antibody (1∶10,000) or anti-mouse antibody (1∶2,000) (both obtained from Pierce, IL, USA).

### Statistics and Data Analysis

The extracted RNA was statistically evaluated to ensure that the groups were not statistically different with respect to the 18 s/28 s rRNA ratio and RNA purity. No differences were found between the groups (data not shown).

For real-time qPCR data normalization, we first measured mRNA levels for the reference genes. Stability comparison of the expression of the reference genes was conducted with the Normfinder software (http://www.mdl.dk). CycA and Rpl13A were determined to be the best combination in the dorsal hippocampus, whereas CycA and Hprt1 were determined to be the best combination in the ventral hippocampus. Therefore, values from each individual sample were normalized with either the geometric mean of the reference genes CycA and Rpl13A or CycA and Hprt1 in the dorsal and ventral hippocampus, respectively. The normalized data are given in Fig. 5.

Statistical analyses were performed with GraphPad Prism version 5.01 for Windows (GraphPad software, San Diego, CA, USA). Group means were analyzed for statistical significance using two-way ANOVA followed by Bonferronìs multiple comparison test. P<0.05 was considered statistically significantly different.
